# Adaptive responses of pear cultivars to low-temperature stress in the spring period

**DOI:** 10.18699/vjgb-26-44

**Published:** 2026-05

**Authors:** A.E. Mishko, A.V. Klyukina

**Affiliations:** North Caucasian Federal Scientific Center of Horticulture, Viticulture, Winemaking, Krasnodar, Russia; North Caucasian Federal Scientific Center of Horticulture, Viticulture, Winemaking, Krasnodar, Russia

**Keywords:** pear cultivars, low-temperature stress, resistance, antioxidant system defense, gene expression, груша, холодовой стресс, устойчивость, антиоксидантная система защиты, экспрессия генов

## Abstract

The pear is one of the most famous pome crops. It occupies about 7 % of the total area of perennial fruit crops in Russia. Orchard plantings are predominantly composed of foreign European cultivars. Spring frosts, which are typical for the southern regions of the country, lead to significant crop losses. This study determined the response characteristics of pear flower buds to low-temperature stress. The Crimean cultivar Dzhankoyskaya Pozdnyaya, two cultivars – Leven and Flamenco – of Krasnodar selection and the interspecific hybrid Kieffer were investigated. Flower buds at different developmental stages were exposed to a climatic chamber for 12 hours at temperatures –1.5…–2 °C. After stress exposure, the activity of certain antioxidant enzymes was determined, along with the content of phenolic compounds, malondialdehyde, and the gene expression level of its enzymes and proteins involved in cold adaptation. It was revealed that the autumn-ripening cultivar Kieffer, under conditions of the Krasnodar region, begins to bloom earlier than other studied cultivars, making it more susceptible to recurrent frosts. This is evidenced by high values of malondialdehyde and the activity level of superoxide dismutase. The Russian cultivars, Leven (winter cultivar) and Flamenco (summer cultivar), showed the highest activity of peroxidase and gene expression of PcDREB2, PcCAP160, PcCOR413, PcPOX1, with a reduced level of malondialdehyde. These cultivars typically emerged from dormancy later compared to Kieffer. The Crimean winter-ripening cultivar was closer to the interspecific hybrid in terms of the studied parameters but showed lower enzyme activity and gene expression levels. The obtained results suggest that under pear cultivation conditions in the southern region of the country, where spring frosts are possible, cultivars with flowering starting in the second-to-third decade of April and high indicators of antioxidant enzyme activity (primarily peroxidase) and gene expression levels of PcDREB2, PcCAP160, and PcCOR413 demonstrate greater resistance.

## Abstract (ru)

Хорошо известная семечковая культура – груша – на территории России занимает около 7 % общих площадей насаждений многолетних плодовых культур. Основную долю грушевых садов составляют зарубежные европейские сорта. Возвратные заморозки, которые характерны для южных регионов страны в весенний период, приводят к значительным потерям урожая. В настоящей работе изучены особенности ответных реакций цветочных почек сортов груши на низкотемпературный стресс. Исследовали крымский сорт Джанкойская поздняя, два краснодарских сорта Левен и Фламенко, а также межвидовой гибрид Киффер. Цветочные почки на разных стадиях развития промораживали в климатической камере в течение 12 ч при температуре –1.5…–2.0 °С. После стресса определяли активность некоторых ферментов антиоксидантной системы, содержание фенольных соединений, малонового диальдегида и уровень экспрессии генов тех же ферментов и белков, участвующих в холодовой адаптации. Выявлено, что осенний сорт Киффер в условиях Краснодарского края быстрее других изученных сортов начинает цвести, вследствие чего наиболее подвержен воздействию возвратных заморозков, о чем свидетельствуют высокие значения малонового диальдегида и уровень активности супероксиддисмутазы. Отечественные сорта Левен (зимний сорт) и Фламенко (летний) обладали самыми высокими показателями активности пероксидазы и экспрессии генов PcDREB2, PcCAP160, PcCOR413, PcPOX1 на фоне сниженного уровня малонового диальдегида. Как правило, данные сорта позднее выходили из состояния покоя по сравнению с сортом Киффер. Крымский сорт зимнего срока созревания был ближе по исследуемым параметрам к межвидовому гибриду, но отличался меньшими показателями ферментативной активности и экспрессией рассматриваемых генов. Полученные результаты позволяют заключить, что в условиях произрастания груши на территории южного региона страны, где возможны возвратные заморозки в весенний период, большей физиологической устойчивостью обладают сорта с началом цветения во второй-третьей декадах апреля, характеризующиеся высокими показателями антиоксидантной ферментативной активности, в первую очередь пероксидазы, и уровнем экспрессии генов PcDREB2, PcCAP160 и PcCOR413.

## Introduction

Pome crops, and pears in particular, are a valuable resource for the horticulture of the Krasnodar region. The share of pears in the total plantings of perennial fruits is extremely small and amounts to about 7 % of the total, though the economic interest in the crop keeps growing. The reasons for its limited distribution, even under the most favorable conditions of southern Russia, are obvious: compared to apple trees, pears are more demanding of growing conditions, mainly water supply, especially at the beginning of the growing season and throughout fruit ripening (Asayesh et al., 2023). The frost resistance of the crop is high: according to various researchers, in a state of organic dormancy, pear flower buds can withstand temperatures up to –25 °C (Sotnik et al., 2017; Bandurko, 2024). Not only sub-zero temperatures and frost duration are critical, but also the phase of the development of generative organs (Xiao et al., 2022).

One of the essential features of adaptation to growing conditions is the resistance of fruit crop cultivars to low-temperature stress during the growing season. In the Krasnodar region, late-spring frosts, which have become more common recently, cause significant economic losses. Based on the long-term weather monitoring, the main stress factors for pears during the winter–spring period have been revealed: the absence of low temperatures in December–January that are necessary for plant hardening, and frosts at the beginning of the growing season (March–April). Each of these stress factors might lead to a reduction in yield or a complete loss of harvest in pear cultivars (Evers et al., 2021; Lee et al., 2023; Zhao et al., 2023). It has been shown that any crop – and any particular cultivar – has its own threshold of critical minimum temperatures that may trigger the death of flower buds (Klyukina et al., 2024). Moreover, different cultivars are characterized by different timeframes of the deep dormancy period: its duration under certain temperature conditions will determine the speed of dormancy release (Gabay, Flaishman, 2024).

The resistance of cultivars to low-temperature stress is composed of the complex interaction of physiological, biochemical, and molecular genetic processes that are reflected in a number of indicators. Any significant stress experienced by a plant provokes secondary oxidative stress caused by an increase in reactive oxygen species (ROS) in cells (Suzuki et al., 2012). One of the key protective reactions to such a negative impact is the increased activity of antioxidant enzymes that inhibit the accumulation of free radicals, which might lead to the destruction of lipids, proteins, and nucleic acids, to the point of cell death. Superoxide dismutase neutralizes superoxide anion, while peroxidases neutralize hydrogen peroxide (Dumanovic et al., 2021). Polyphenol oxidase catalyzes the oxidation reactions of various phenolic compounds, synthesizing or degrading the metabolites necessary for maintaining plant homeostasis (Zhang S., 2023).

Along with enzymes, numerous phenolic compounds also perform a protective function (Dumanovic et al., 2021). Flavonoids are one of the main phenolic antioxidants. They are capable of reducing ROSs to stable and less harmful forms (Treml, Mejkal, 2016). At the genetic level, low-temperature stress induces the expression of functional and regulatory genes, the products of which either directly protect cells from damage (osmolite biosynthesis enzymes, detoxification enzymes, etc.) or participate in signal transduction and the regulation of gene expression (Yang, Huang, 2018; Zhang Y. et al., 2023).

Due to the increased frequency of late frosts in the Krasnodar region, the aim of this research is to study the response of pear cultivars to low-temperature stress during the spring season.

## Materials and methods

The survey was conducted in the Kuban horticultural zone of the Krasnodar region (45°16′N, 38°93′E) in March–April 2023–2025 on the genetic collection of the North Caucasian Federal Scientific Center of Horticulture, Viticulture, Winemaking (NCFSCHVW). The objects of the study were the pear (Pyrus communis L.) cultivars of domestic origin Leven and Flamenco (NCFSCHVW breeding), the Crimean cultivar Dzhankoyskaya Pozdnyaya and the American cultivar Kieffer (an interspecific hybrid of P. communis × P. pyrifolia Nakai). The cultivars were grafted onto the BA-29 rootstock. The planting year was 2007, and the planting pattern was 5 × 2 m. Ten shoots with flower buds were selected from 5–7 trees of each cultivar.

The response of generative organs to low spring temperatures was analyzed by the critical minimum temperatures for each phase of flower bud development in the BPC500D/ CVSI-Spector climate chamber (Fujian Jiupo Biotechnology Co, China) using the standard methodology (Program and Methodology of Varietalization…, 1999; New Methods…, 2023). Phases of flower bud development were determined according to the generally accepted techniques (Kolomiyts, 1952; Shitt, 1958). The temperature data for the studied period were obtained from a local weather station (synoptic index 34927). Natural late frosts were simulated under laboratory conditions, with a gradual decrease in temperature to a critical level of –1.5 to –2.0 °C over 24 hours. Control samples were not exposed to low-temperature stress. Upon completing the experiment, the plant material was frozen in liquid nitrogen for further use in a series of biochemical and molecular genetic analyses

The total content of phenolic compounds and flavonoids was determined in pear flower buds (Ainsworth, Gillespie, 2007; Hikmawanti et al., 2021). The extraction of soluble proteins was conducted using the method outlined by Z. Wei and colleagues (2018). The concentration of soluble proteins was assessed by coloring with Coomassie solution (Bradford, 1976). The activity of superoxide dismutase (SOD), peroxidase (POX), and polyphenol oxidase (PPO) enzymes was measured according to standard colorimetric methods (Boyarkin, 1951; Queiroz et al., 2011; Efimova et al., 2018). The content of malondialdehyde (MDA) was identified by the reaction with thiobarbituric acid (Bonyanpour, Jamali, 2020).

To analyze gene expression levels, RNA was extracted from pear flower buds using a modified CTAB method (Sundyreva et al., 2018). cDNA synthesis was carried out using MMLV reverse transcriptase (Eurogen, Russia) according to the manufacturer’s instructions. Real-time PCR was conducted by means of the qPCRmix-HS SYBR kit (Eurogen, Russia). The primers were selected from literature sources (Table 1). A conservative fragment of the actin gene sequence was used as a reference gene. The relative expression of the studied genes was calculated via the 2–ΔΔCt method (Livak, Schmittgen, 2001).

**Table 1.**
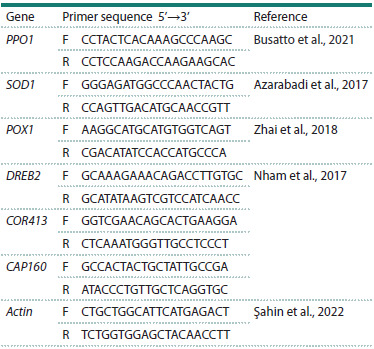
Sequences for primers used in quantitative real-time PCR Note. F – forward primer sequence, R – reverse primer sequence.

Measurements were taken in 2–4-fold analytical repetition. The statistical significance of differences was determined based on the results of the Tukey test of one-way ANOVA at the 0.05 significance level. Statistical analysis was carried out using the STATISTICA 12 software. The results are presented as mean values and their standard errors.

## Results

**Fig. 1.**
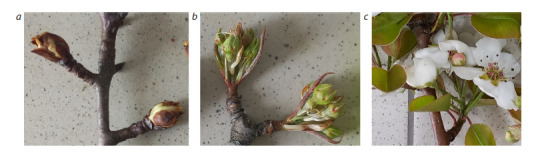
Phases of pear flower bud development a – bud burst; b – flower bud separation; c – start of blossoming.

The temperature conditions during the study period were variable (Table 2), as can be seen in the phases of flower bud development (Fig. 1). The plant material was selected in the third decade of March or the first decade of April, when the pear starts blossoming.

**Table 2.**
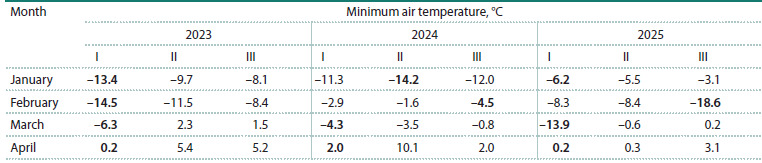
Temperature regime in the winter-spring period of 2023–2025 Note. I–III – decades of the months; the absolute minimum temperatures for the month are shown in bold.

The conditions for bursting of generative buds were favorable in 2023. After spring frosts in March (down to –6.3 °C), the temperature grew up to +5.4 °C in April, and this resulted in the early start of blossoming for the Kieffer cultivar, while Flamenco, Leven, and Dzhankoyskaya Pozdnyaya remained in the phase of flower bud separation.

The development of generative organs in 2024 was different from that in 2023. Temperatures in March dropped as low as –4.3 °C, while in April the minimum temperature was only +2.0 °C. Most of the cultivars (Leven, Dzhankoyskaya Pozdnyaya, and Flamenco) remained in the phase of bud burst, whereas Kieffer was in the phase of flower bud separation.

Over the three years of the study, the lowest temperature was recorded in March (down to –13.9 °C) only in 2025. The minimum air temperature in the first ten days of April was +0.2 °C. Under these conditions, it was found that Dzhankoyskaya Pozdnyaya and Flamenco were in the phase of bud burst, while Kieffer and Leven reached the phase of flower bud separation.

**Fig. 2.**
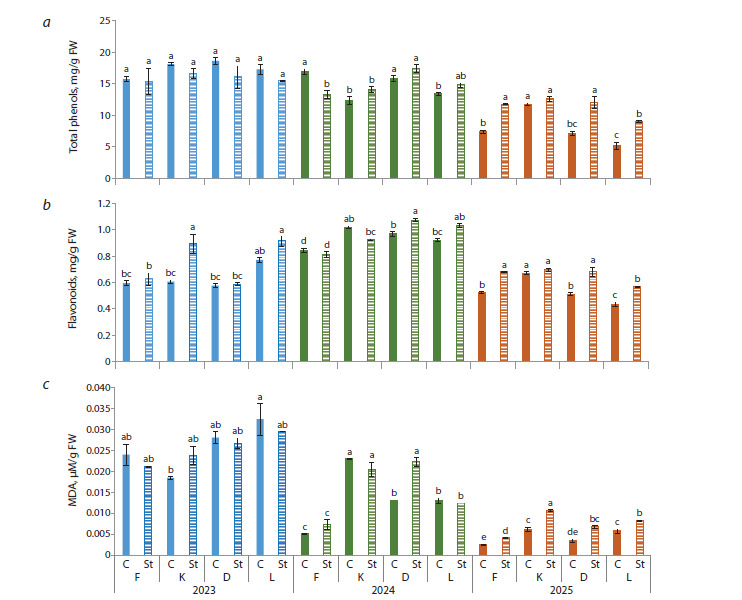
Content of total phenols, flavonoids, malondialdehyde in pear flower buds under low-temperature stress conditions. Here and in Figures 2–5: F – Flamenco, K – Kieffer, D – Dzhankoyskaya Pozdnyaya, L – Leven; C – control, St – stress; 2023–2024 – years; significant differences of the data are shown by different lowercase letters based on the results of the Tukey test at p ≤ 0.05.

The total phenolic content in pear flower buds varied in different years of the study (Fig. 2a). The maximum values for most cultivars were recorded in 2023, averaging from 17.2 to 18.6 mg/g FW. The minimum values were registered in the spring of 2025: 5.1–11.7 mg/g FW. Upon short-term lowtemperature stress, a significant increase in this parameter was observed only in 2025. Flamenco, Dzhankoyskaya Pozdnyaya, and Leven had almost a twofold increase in phenols. Throughout the study period, Leven on average was characterized by the lowest values of this parameter, i. e. ⁓12 mg/g FW.

With respect to the flavonoid content, the highest values were recorded in 2024, ranging from 0.8 to 1.1 mg/g FW (Fig. 2b). In 2025, the accumulation of flavonoids in pear flower buds reached its minimum (0.4–0.6 mg/g FW). Due to the stress, the flavonoid content increased by more than 30 % in 2025, except for Kieffer, which surged by 1.5 times in 2023. Thus, during the three-year experiment, the maximum levels of flavonoids after stress were found in different cultivars: in 2023, in Kieffer and Leven; in 2024, in Dzhankoyskaya Pozdnyaya and Leven; and in 2025, in Flamenco, Dzhankoyskaya Pozdnyaya, and Kieffer.

Significant differences in the malondialdehyde accumulation were found depending on the year of the study. The highest values were specific to 2023, averaging 0.03 μM/g FW, and the lowest values were observed in 2025, averaging 0.004 μM/g FW (Fig. 2c). Low-temperature stress caused an increase in MDA by 1.5–2 times for all pear cultivars in 2025; for Flamenco and Dzhankoyskaya Pozdnyaya, by 45 and 65 %, respectively, in 2024; and by 30 %, for Kieffer in 2023. The analysis of the mean values of this parameter for the 2023–2025 period showed the maximum stress values in Leven, Dzhankoyskaya Pozdnyaya, and Kieffer.

**Fig. 3.**
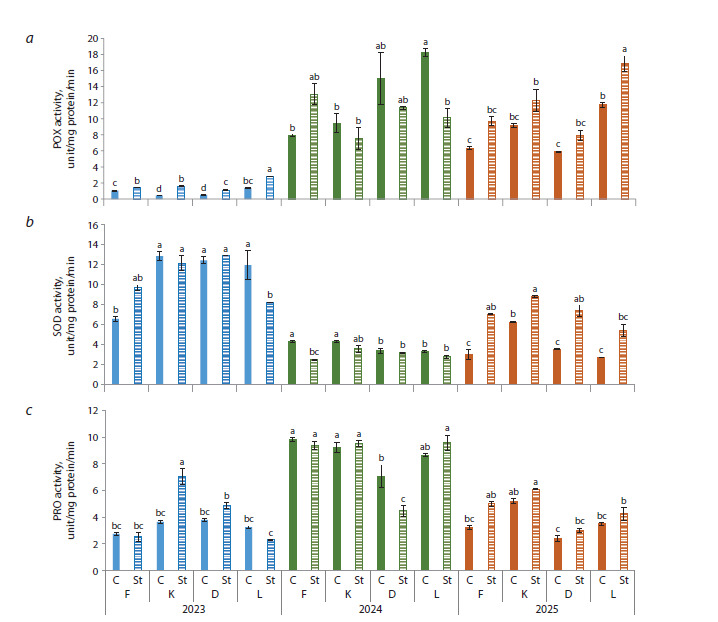
Activities of peroxidase, superoxide dismutase, polyphenol oxidase in pear flower buds under low-temperature stress conditions in 2023–2025.

Changes in the activity of the studied enzymes also depended on the year of the study. Peroxidase activity reached its maximum in the springs of 2024–2025 (from 5.9 to 18.3 unit/mg protein/min), and its minimum in 2023, ranging from 0.5 to 1.4 unit/mg protein/min (Fig. 3a). Most times stress led to an increase in POX activity, in some cases more than twofold. The highest enzyme activity levels were observed in Leven after exposure to low air temperatures under simulated conditions, averaging 11.6 units/mg protein/min over the entire study period.

The dynamics of superoxide dismutase activity were opposite to the changes of peroxidase activity, with peak growth in 2023 and its minimum in 2024. Throughout the study period, the highest SOD values were recorded in Kieffer, averaging 6.8 unit/mg protein (Fig. 3b). After stress, the enzyme activity in pear flower buds increased by 1.4–2.4 times only in 2025. In the course of three years, the highest values after short-term negative effects were found in Kieffer and Dzhankoyskaya Pozdnyaya, averaging 8.0 unit/mg protein.

The activity of polyphenol oxidase also varied from year to year: the maximum was in 2024, and the minimum was in 2023 and 2025 (Fig. 3c). Cultivar differences were not great; only for Dzhankoyskaya Pozdnyaya PPO values were the lowest in 2024 and 2025, 7.1 and 3.0 unit/mg protein, respectively. Low-temperature stress did not lead to a significant change in enzyme activity, except for two cases: a 2-fold PPO increase in Kieffer in 2023 and a 60 % PPO decrease in Dzhankoyskaya Pozdnyaya in 2024.

**Fig. 4.**
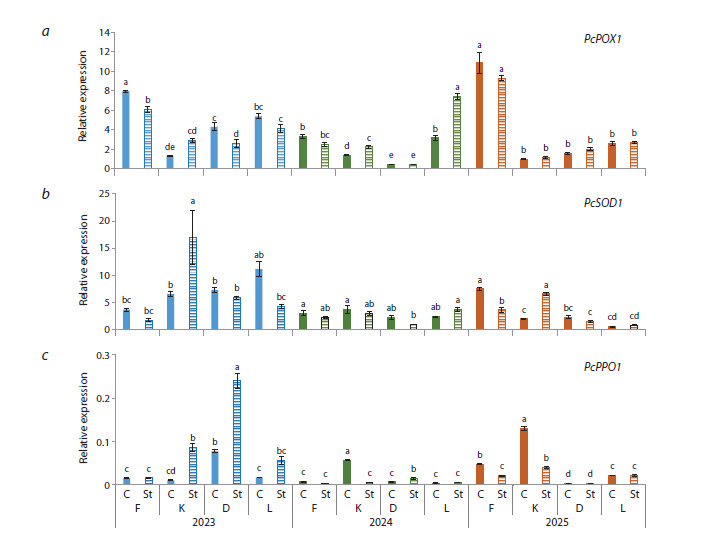
Relative expression of the PcPOX1, PcSOD1, PcPPO1 genes in pear flower buds under low-temperature stress conditions in 2023–2025.

According to the level of relative gene expression of the same antioxidant enzymes, the highest mean values of PcPOX1 and PcSOD1 were found in 2023 (Fig. 4a, b). Meanwhile, the maximum relative expression of the peroxidase gene was recorded for Kieffer in 2025. Regarding the PcPPO1 gene, a high level of expression was observed in 2025, with a maximum in Kieffer (Fig. 4c). Subsequent to the stress, the expression level of antioxidant enzyme genes did not change significantly, except for certain cases: a spike in PcSOD1 was observed in Kieffer in 2023 and 2025; PcPOX1 also increased in Leven in 2024; a decrease was recorded in PcPOX1 for Flamenco and Dzhankoyskaya Pozdnyaya in 2023, then in PcSOD1 for Flamenco in 2025, and in PcPPO1 for Kieffer in 2024–2025 (in 2023, on the contrary, an increase in this parameter was noted).

**Fig. 5.**
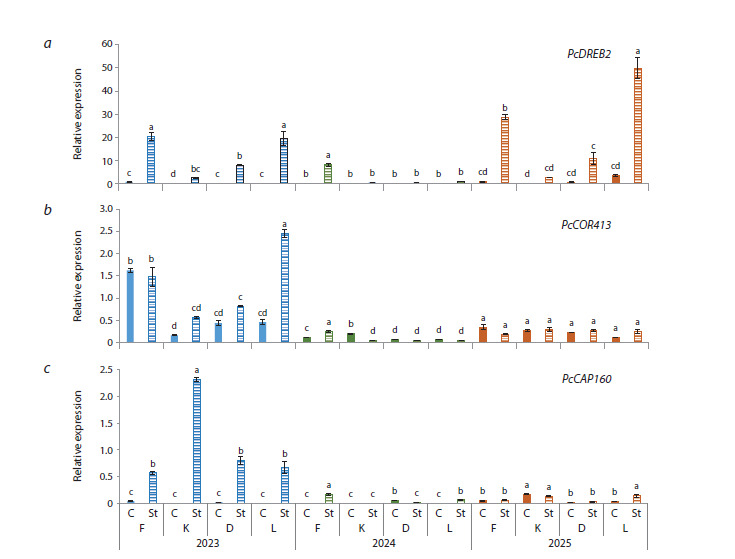
Relative expression of the PcDREB2, PcCOR413, PcCAP160 genes in pear flower buds under low-temperature stress conditions in 2023–2025.

Genetic analysis was also conducted for a group of genes responsible for resistance to low-temperature stress: the transcription factor PcDREB2 (Fig. 5a) and the proteins of cold acclimation PcCAP160, PcCOR413 (Fig. 5b, c) were studied. No cultivar differences were found under the control conditions throughout the study, except for PcCOR413 in Flamenco in 2023, which exceeded the values of the others by 78 %.

The exposure of pear generative buds to low temperatures caused a significant increase in the level of relative expression for PcCOR413 – by 2–5 times in 2023 alone for all the studied cultivars. In Flamenco, an increase in stress values of this gene was detected in 2024 (by two times), since in the first year of the study, as noted earlier, the reference indicators were quite high. As for the level of relative expression for PcCAP160, its values under stressful conditions surged in 2023, especially in Kieffer, as well as in Flamenco and Leven in 2024 by 8–14 times, and in the latter cultivar, by three times in 2025. The changes in indicators for PcDREB2 were even more dynamic. In 2023, the reference values were exceeded by 30–46 times for Flamenco and Dzhankoyskaya Pozdnyaya, by 109 times for Leven, and by 122 times for Kieffer with minimal values. In the following year, the growth of PcDREB2 indicators was not so significant – by 2–17 times, though the expression level in Flamenco increased by 68 times. The maximum values for PcDREB2 were recorded after the stress in Flamenco and Leven in 2025. An increase in expression, but less significant, was also registered in Kieffer and Dzhankoyskaya Pozdnyaya.

## Discussion

According to the data obtained, it was revealed that physiological, biochemical, and molecular genetic characteristics of the generative bud are defined by its developmental stage. The “bud burst” stage is characterized by a high activity of POX and PPO enzymes, as well as accumulation of flavonoids. Under colder conditions in the spring of 2025, at the same development stage of the generative structures, a more pronounced stress response was observed, taking the form of increased activity of peroxidase and superoxide dismutase, more intense accumulation of total phenols, flavonoids, MDA, and high expression of the PcDREB2 gene. Similar results regarding the increase in antioxidant enzyme activity during the budding of pear P. pyrifolia were presented in the work by S. Hussain and colleagues (2015).

The “flower bud separation” stage is defined by the maximum accumulation of total phenols and MDA, the highest SOD activity, and a relatively high level of PcCOR413 expression. Under stress, POX activity increased, and the expression of the PcPPO1, PcDREB2, PcCOR413 and PcCAP160 genes spiked. The “start of blossoming” stage, which was recorded only in Kieffer in 2023, is characterized by a more intense response to stress, including increased synthesis of flavonoids, PPO activity, and high levels of expression for all studied genes. When studying the response of flowers of different Asian pear genotypes to low-temperature stress from +2 to –4 °C for four hours, Chinese researchers observed an increase in SOD activity and in the proportion of gene transcripts related to flavonoid biosynthesis (Li et al., 2023; Lin et al., 2023). In another study, exposure of apple cell culture to low temperatures led to the activation of DREB/CBF gene expression (Du et al., 2015).

As shown above, the expression level of the PcDREB2 gene in pear flower buds after stress was characterized by maximum growth (up to 100-fold increase), which corresponds to the data on the Ussuri pear (P. ussuriensis Maxim. ex Rupr.) – with the highest expression of the DREB1 and DREB2 genes in the first 12 hours under hypothermia (Yang, Huang, 2018). At the same time, in the fruits of the Williams cultivar, under exposure to low temperatures, no increase was detected in the expression of the CBF1, CBF4, DREB2, COR413 genes, except for CAP160 (Nham et al., 2017).

Based on the comparative analysis of the studied pear cultivars, it can be concluded that the autumn cultivar Kieffer has the ability to quickly emerge from a state of deep dormancy, which determines its earlier flowering period. As noted by G. Gabay and M.A. Flaishman, this is linked with the cultivar origin, since one of its parent forms is P. pyrifolia, which does not require low temperatures to enter a deep dormancy phase and quickly emerges from it after warm winters (Gabay, Flaishman, 2018). The relatively high stress levels of MDA are likely to contribute to its increased susceptibility to low temperatures during spring, resulting in a rapid response. Dzhankoyskaya Pozdnyaya, a winter-ripening cultivar, like Kieffer, is defined by a high content of phenolic compounds, as well as an increase in MDA content and SOD activity under stress conditions, but a less pronounced antioxidant defense system and lower expression of cold resistance genes. Although Flamenco is a summer-ripening cultivar and Leven is winter-ripening, both cultivars had similar responses to low-temperature stress. On average, SOD activity and MDA content were at or below the reference values during the entire study period, while POX values and the expression of the PcPOX1, PcDREB2, PcCAP160 and PcCOR413 genes reached their maximum.

## Conclusion

The conducted study revealed a difference in how pear cultivars of various ripening period and origin adapt to low-temperature stresses during winter and spring both at the ecological and at the physiological level. Pear plants with earlier flowering, such as Kieffer, are more susceptible to low temperatures due to increased SOD activity and high levels of MDA accumulation. In cultivars with later flowering (II–III decade of April), the protective mechanisms for containing oxidative stress in plant cells were activated more quickly: the maximum expression levels of cold resistance genes and peroxidase activity were observed at low MDA levels.

The obtained results will enable further evaluation of pear cultivars by cold resistance markers in order to create the latest inventory of promising cultivars and ensure their rational distribution in the southern regions of Russia that will result in stable and high-quality yields.

## Conflict of interest

The authors declare no conflict of interest.

## References

Ainsworth E.A., Gillespie K.M. Estimation of total phenolic content
and other oxidation substrates in plant tissues using Folin–Ciocalteu
reagent. Nat Protoc. 2007;2:875-877. doi 10.1038/nprot.2007.102

Asayesh Z.M., Arzani K., Mokhtassi-Bidgoli A., Abdollahi H. Enzymatic
and non-enzymatic response of grafted and ungrafted young
European pear (Pyrus communis L.) trees to drought stress. Sci
Hortic.
2023;310:111745. doi 10.1016/j.scienta.2022.111745

Azarabadi S., Abdollahi H., Torabi M., Salehi Z., Nasiri J. ROS generation,
oxidative burst and dynamic expression profiles of ROSscavenging
enzymes of superoxide dismutase (SOD), catalase (CAT )
and ascorbate peroxidase (APX ) in response to Erwinia amylovora
in pear (Pyrus communis L.). Eur J Plant Pathol. 2017;147:279-294.
doi 10.1007/s10658-016-1000-0

Bandurko I.A. Assessment of the gene pool of the Pear by resistance to
adverse factors of the winter period in the foothill zone of the North-
West Caucasus. Science and Innovation. 2024;21:88-92 (in Russian)

Bonyanpour A.R., Jamali B. Seasonal enzymatic and non-enzymatic
antioxidant responses in seven Iranian pomegranate cultivars. Adv
Hortic Sci. 2020;34(3):265-276

Boyarkin A.N. A quick method for determining the activity of peroxidase.
Biochemistry. 1951;16(4):352-355 (in Russian)

Bradford M.M. A rapid and sensitive method for the quantitation of microgram
quantities of protein utilizing the principle of protein dye
binding. Anal Biochem. 1976;72(1-2):248-254. doi 10.1016/0003-
2697(76)90527-3

Busatto N., Giné-Bordonaba J., Larrigaudière C., Lindo-Garcia V., Farneti
B., Biasioli F., Vrhovsek U., Costa F. Molecular and biochemical
differences underlying the efficacy of lovastatin in preventing the
onset of superficial scald in a susceptible and resistant Pyrus communis
L. cultivar. Postharvest Biol Technol. 2021;173:111435. doi
10.1016/j.postharvbio.2020.111435

Du F., Xu J.-N., Li D., Wang X.-Y. The identification of novel and differentially
expressed apple-tree genes under low-temperature stress
using high-throughput Illumina sequencing. Mol Biol Rep. 2015;42:
569-580. doi 10.1007/s11033-014-3802-5

Dumanovic J., Nepovimova E., Natic M., Kuca K., Jacevic V. The significance
of reactive oxygen species and antioxidant defense system
in plants: a concise overview. Front Plant Sci. 2021;11:552969. doi
10.3389/fpls.2020.552969

Efimova M.V., Kolomeichuk L.V., Boyko E.V., Malofii M.K., Vidershpan
A.N., Plyusnin I.N. Golovatskaya I.F., Murgan O.K., Kuznetsov
Vl.V. Physiological mechanisms of Solanum tuberosum L.
plants’ tolerance to chloride salinity. Russ J Plant Physiol. 2018;
65(3):394-403. doi 10.1134/S1021443718030020

Evers S.M., Knight T.M., Inouye D.W., Miller T.X., Salguero-Gómez R.,
Iler A.M., Compagnoni A. Lagged and dormant season climate better
predict plant vital rates than climate during the growing season. Glob
Chang Biol. 2021;27(9):1927-1941. doi 10.1111/gcb.15519

Gabay G., Flaishman M.A. Genetic and molecular regulation of chilling
requirements in pear: breeding for climate change resilience. Front
Plant Sci. 2024;15:1347527. doi 10.3389/fpls.2024.1347527

Hikmawanti N., Fatmawati S., Asri A.W. The effect of ethanol concentrations
as the extraction solvent on antioxidant activity of katuk
(Sauropus androgynus (L.) Merr.) leaves extracts. IOP Conf Ser
Earth Environ Sci. 2021;755:012060. doi 10.1088/1755-1315/755/
1/012060

Hussain S., Liu G., Liu D., Ahmed M., Hussain N., Teng Y. Study on the
expression of dehydrin genes and activities of antioxidative enzymes
in floral buds of two sand pear (Pyrus pyrifolia Nakai) cultivars
requiring different chilling hours for bud break. Turk J Agric For.
2015;39(6):930-939. doi 10.3906/tar-1407-164

Klyukina A.V., Dragavtseva I.A., Oplachko R.A. Study of fruit crops
varieties’ needs in temperature regime of their development phases
(on the example of pear varieties). Fruit Growing and Viticulture of
South Russia. 2024;89(5):49-58. doi 10.30679/2219-5335-2024-5-
89-49-58 (in Russian)

Kolomiyts I.A. Biological analysis of the development of flower buds
in an apple tree. Doklady Akademii Nauk SSSR. 1952;84(4):821-824
(in Russian)

Lee J.C., Park Y.S., Jeong H.N., Kim J.H., Heo J.Y. Temperature changes
affected spring phenology and fruit quality of apples grown
in high-latitude region of South Korea. Horticulturae. 2023;9(7):
794. doi 10.3390/horticulturae9070794

Li Y., Zhang J., Wang S., Zhang H., Liu Y., Yang M. Integrative transcriptomic
and metabolomic analyses reveal the flavonoid biosynthesis
of Pyrus hopeiensis flowers under cold stress. Hortic Plant J.
2023;9(3):395-413. doi 10.1016/j.hpj.2022.11.004

Lin S., Li Y., Zhao J., Guo W., Jiang M., Li X., Liu W., Zhang J.,
Yang M. Transcriptome analysis of biochemistry responses to lowtemperature
stress in the flower organs of five pear varieties. Forests.
2023;14(3):490. doi 10.3390/f14030490

Livak K.J., Schmittgen T.D. Analysis of relative gene expression data
using real-time quantitative PCR and the 2–ΔΔCT method. Methods.
2001;25(4):402-408. doi 10.1006/meth.2001.1262

New Methods of Radical Increase in Yields of Fruit Crops Based on the
Theory of the Environmental and Genetic Organization of Quantitative
Features in the Context of Climate Fluctuation. Krasnodar, 2023
(in Russian)

Nham N.T., Macnish A.J., Zakharov F., Mitcham E.J. ‘Bartlett’ pear
fruit (Pyrus communis L.) ripening regulation by low temperatures
involves genes associated with jasmonic acid, cold response,
and transcription factors. Plant Sci. 2017;260:8-18. doi 10.1016/
j.plantsci.2017.03.008

Program and Methodology of Varietalization of Fruit, Berry and Walnut
Crops. Orel, 1999 (in Russian)

Queiroz C., da Silva A.J.R., Lopes M.L.M., Fialho E., Valente-Mesquita
V.L. Polyphenol oxidase activity, phenolic acid composition and
browning in cashew apple (Anacardium occidentale L.) after processing.
Food Chem. 2011;125(1):128-132. doi 10.1016/j.foodchem.
2010.08.048

Şahin Ö., Dumanoğlu H., Sarikamiş G., Javadisaber J., Ergül A.,
Aydemir B.Ç. Tolerance of Pyrus spp. and Cydonia oblonga as pear
rootstocks to iron chlorosis determined by in vitro growth, antioxidant
and molecular responses. Sci Hortic. 2022;296:110911. doi
10.1016/j.scienta.2022.110911

Shitt P.G. The Study of the Growth and Development of Fruit and Berry
Plants. Moscow, 1958 (in Russian)

Sotnik A.I., Babina R.D., Khoruzhy P.G. Comparative assessment of
resistance of the generative organs of pear varieties (Pirus communis
L.) to low-temperature stresses under the conditions of Crimea.
Izvestia Orenburg State Agrarian University. 2017;3(65):72-74 (in
Russian)

Sundyreva M.A., Stepanov I.V., Suprun I.I., Ushakova Y.V. A modified
protocol of RNA isolation from mature leaves of grapes for RT-PCR.
Scientific Journal of Kuban State Agrarian University. 2018;143:16-
30. doi 10.21515/1990-4665-143-012 (in Russian)

Suzuki N., Koussevitzky S., Mittler R., Miller G. ROS and redox signalling
in the response of plants to abiotic stress. Plant Cell Environ.
2012;35(2):259-270. doi 10.1111/j.1365-3040.2011.02336.x

Treml J., Mejkal K. Flavonoids as potent scavengers of hydroxyl
radicals. Compr Rev Food Sci Food Saf. 2016;15(4):720-738. doi
10.1111/1541-4337.12204

Wei Z., Gao T., Liang B., Zhao Q., Ma F., Li C. Effects of exogenous
melatonin on methyl viologen-mediated oxidative stress in apple
leaf. Int J Mol Sci. 2018;19(1):316. doi 10.3390/ijms19010316

Xiao L.J., Asseng S., Wang X.T., Xia J.X., Zhang P., Liu L.L., Tang L.,
Cao W., Zhu Y., Liu B. Simulating the effects of low-temperature
stress on wheat biomass growth and yield. Agric For Meteorol.
2022;326:109191. doi 10.1016/j.agrformet.2022.109191

Yang T., Huang X.-S. Deep sequencing-based characterization of transcriptome
of Pyrus ussuriensis in response to cold stress. Gene.
2018;661:109-118. doi 10.1016/j.gene.2018.03.067

Zhai R., Liu J., Liu F., Zhao Y., Liu L., Fang C., Wang H., Li X.,
Wang Z., Ma F., Xu L. Melatonin limited ethylene production, softening
and reduced physiology disorder in pear (Pyrus communis L.)
fruit during senescence. Postharvest Biol Technol. 2018;139:38-46.
doi 10.1016/j.postharvbio.2018.01.017

Zhang S. Recent advances of polyphenol oxidases in plants. Molecules.
2023;28(5):2158. doi 10.3390/molecules28052158

Zhang Y., Wu L., Liu L., Jia B., Ye Z., Tang X., Heng W., Liu L. Functional
analysis of PbbZIP11 transcription factor in response to cold
stress in Arabidopsis and pear. Plants. 2023;13(1):24. doi 10.3390/
plants13010024

Zhao Y., Hu M.Y., Wang Q., Yan X.K., Lu M.Y., Wu C.H., Li H.Y.,
Zhang M.J. Climate variation and its influence on the cold tolerance
and phenology periods of pear cultivars in Jilin, China. Int J Fruit
Sci. 2023;23(1):165-180. doi 10.1080/15538362.2023.2249995

